# Frequent falls and confusion: recurrent hypoglycemia in a patient with tuberous sclerosis complex

**DOI:** 10.1002/ccr3.1483

**Published:** 2018-03-24

**Authors:** Alexander N. Comninos, Lisa Yang, Ali Abbara, Waljit S. Dhillo, J.H. Duncan Bassett, Jeannie F. Todd

**Affiliations:** ^1^ Department of Endocrinology Hammersmith Hospital Imperial College Healthcare NHS Trust Du Cane Road London W12 0HS UK; ^2^ Section of Endocrinology & Investigative Medicine Department of Medicine Imperial College London Du Cane Road London W12 0HS UK; ^3^ Molecular Endocrinology Laboratory Department of Medicine Imperial College London Du Cane Road London W12 0HS UK

**Keywords:** Confusion, falls, hypoglycemia, insulinoma, tuberous sclerosis complex

## Abstract

Recurrent hypoglycemia is common, but its presentation is often insidious resulting in delays in diagnosis and significant morbidity. We describe a case of an insulinoma presenting with falls and confusion in a patient with tuberous sclerosis, demonstrating the importance of early hypoglycemia identification and a potential shared molecular pathogenesis.

## Introduction

Hypoglycemia is a common and life‐threatening condition. Hypoglycemic symptoms may be subtle and highly variable, ranging from autonomic dysfunction (e.g., sweating and palpitations) to neuroglycopenic effects including behavioral changes, confusion, and seizures. Untreated, severe progressive hypoglycemia is associated with significant morbidity and mortality, and therefore, clinical guidelines have been developed to aid the clinician 1.

Despite their low incidence, insulinomas are the most frequent neuroendocrine tumor of the pancreas and the most common cause of endogenous hyperinsulinemia 1. Tuberous sclerosis complex (TSC) is an autosomal dominant condition characterized by the presence of multiple hamartomas 2. An association between TSC and insulinomas has been reported in a limited number of case reports since 1959 3, 4, 5, 6, 7. Here, we describe a case of recurrent hypoglycemia in a patient with TSC presenting as frequent falls and confusion.

This case highlights the difficulties in recognizing the clinical manifestations of hypoglycemia. The majority of patients with TSC have some cognitive impairment, behavioral abnormalities, or seizures; therefore, distinguishing the clinical manifestations of hypoglycemia was particularly challenging in this patient. We also summarize the diagnostic workup for hypoglycemia and a suspected insulinoma (highlighting the relevant guidelines 1, 8, 9, 10, 11), with appropriate images. Furthermore, we postulate a common pathophysiological mechanism underlying the association between TSC and insulinoma.

## Case Presentation

**Figure 1:**
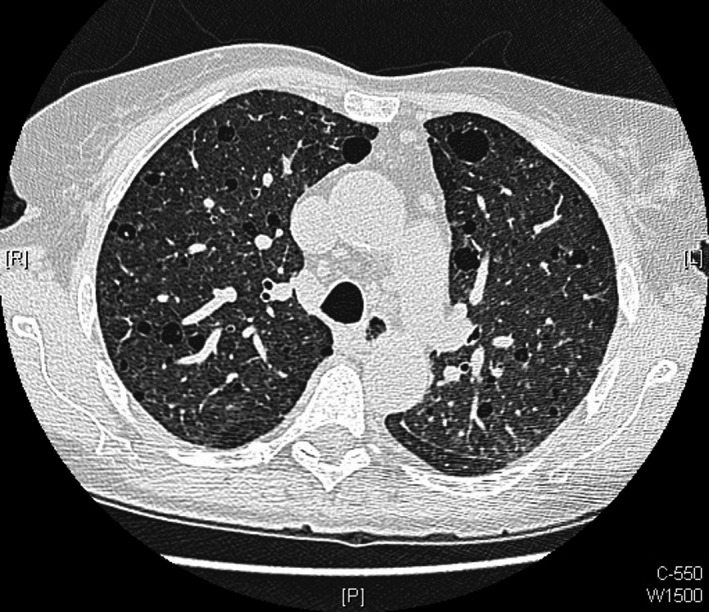
High‐resolution computed tomography (HRCT) chest showing progressive replacement of lung parenchyma with multiple cysts, and proliferation of abnormal smooth‐muscle cells, in a process termed lymphangioleiomyomatosis (LAM).

**Figure 2:**
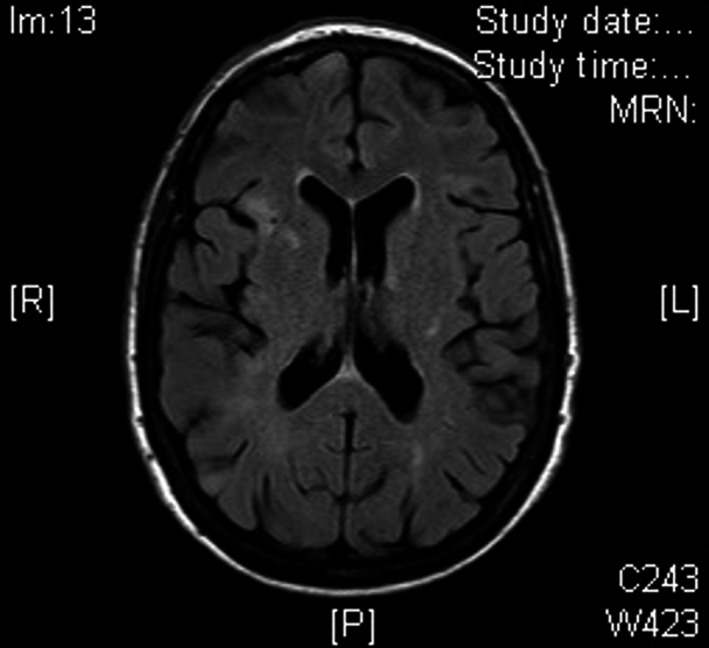
Magnetic resonance imaging (MRI) brain FLAIR sequence showing multiple cortical and subcortical white matter lesions in both cerebral hemispheres consistent with cerebral tubers.

A 67‐year‐old Caucasian woman was admitted to hospital following an episode of confusion and slurred speech during hemodialysis. She had a past medical history of TSC, diagnosed aged 47 years when she presented with breathlessness, with high‐resolution computed tomography (HRCT) of her chest revealing multiple cysts and proliferation of abnormal smooth muscle (Fig. 1). In addition, she developed seizures, with magnetic resonance imaging (MRI) of her brain demonstrating multiple cortical and subcortical white matter lesions consistent with cerebral tubers (Fig. 2). Subsequently, she was found to have large bilateral renal angiomyolipomas, ultimately requiring bilateral nephrectomies for malignant transformation. She commenced regular hemodialysis three times per week, aged 60 years.

Over the previous 2 years (from the age of 65), she had suffered worsening cognition, multiple episodes of confusion with slurred speech, and falls resulting in fractures to her wrist, fingers, and right pelvis. She had also gained 3 kg in weight during the preceding 6 months. Hypoglycemia had been documented by paramedics on previous admissions, but she had not received any specialist endocrine evaluation prior to this presentation. She was not on any medications known to cause hypoglycemia. Her sister and son had also been diagnosed with TSC, but there was no other past medical or family history of note. She stopped smoking 35 years previously and consumed alcohol only occasionally.

**Figure 3:**
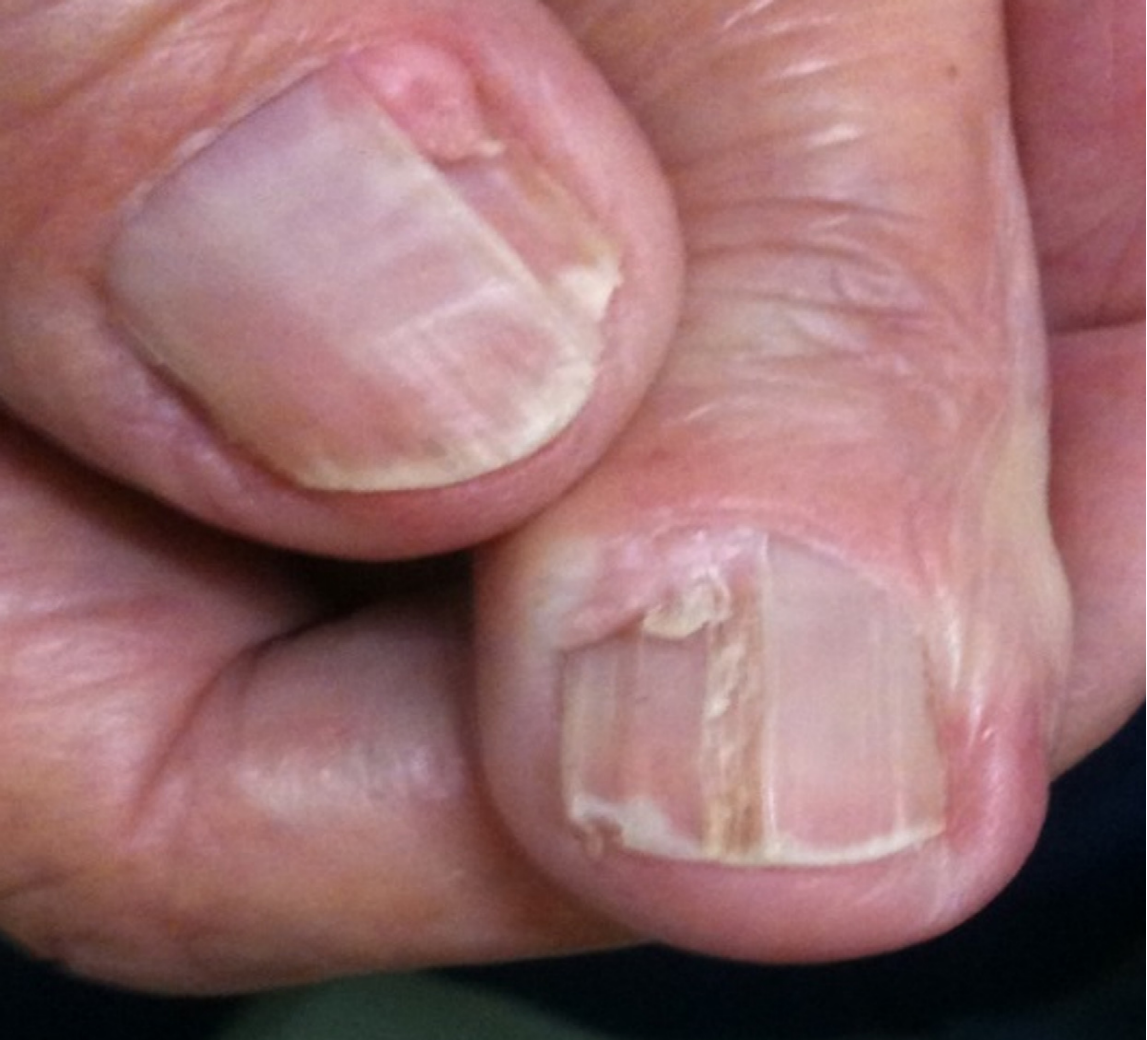
Periungual fibromas (Koenen's tumors) growing around and under nails.

On assessment, she displayed typical cutaneous features of TSC including periungual fibromas (Fig. 3) and facial angiofibromas. Examination and initial investigations were otherwise unremarkable aside from a borderline low‐venous glucose of 3.3 mmol/L. Over the next 48 h, she suffered several episodes of symptomatic hypoglycemia which were not related to food intake. Symptoms were relieved with oral glucose, thus satisfying Whipple's triad to confirm clinical hypoglycemia: (1) typical clinical symptoms of hypoglycemia, (2) low‐circulating glucose concentration, and (3) disappearance of symptoms after the administration of glucose. An endocrine review was therefore requested during this admission.

Routine blood tests (including calcium and other electrolytes) were noncontributory, and CT brain showed no acute cause for her presentation. Important differential diagnoses causing hypoglycemia were excluded as follows. There was no evidence of sepsis, and liver function was normal. Anterior pituitary function profile was normal, including IGF‐1 levels which excluded growth hormone deficiency as a cause. A short synacthen test showed an appropriate peak cortisol of 706 nmol/L excluding adrenal insufficiency. In accordance with the Endocrine Society Guidelines on the Management of Adult Hypoglycemia 1, a supervised 72‐h fast was carried out. This was commenced after dinner subsequent to a dialysis session. The test was terminated after 12 h when the patient developed symptomatic hypoglycemia (venous glucose 1.7 mmol/L, NR >3.0 mmol/L) with an inappropriately elevated insulin (5.9 mU/L, NR <3.0 mU/L) and C‐peptide (3906 pmol/L, NR <300 pmol/L). C‐peptide was not interpretable as she had end‐stage renal failure. However, inappropriately raised insulin levels in the face of hypoglycemia, associated with negative blood ketones (due to ketogenesis suppression by raised insulin), and negative sulphonylurea screens (excluding surreptitious consumption of sulphonylurea medication) confirmed a diagnosis of endogenous hyperinsulinemia 1.

**Figure 4:**
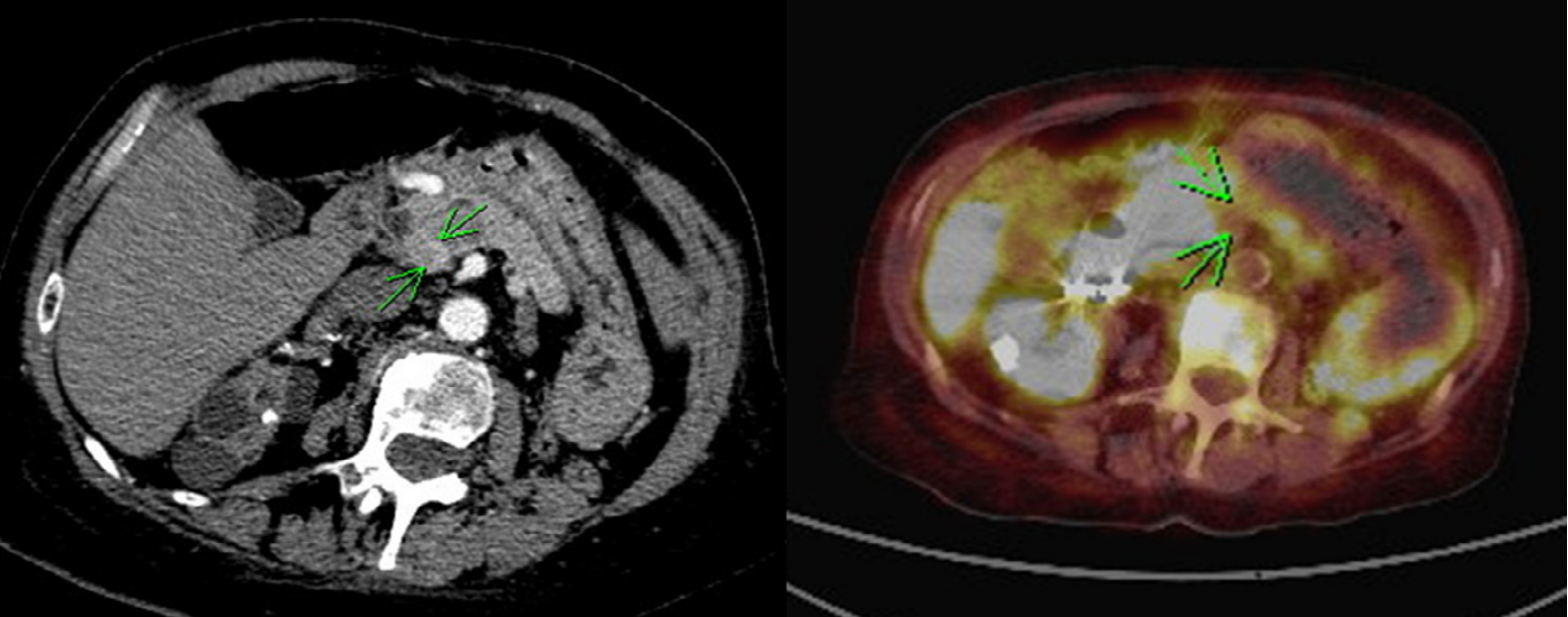
Abdominal computed tomography scan (CT) following a pancreatic protocol (left) and Gallium DOTATATE positron emission tomography/computed tomography scan (PET/CT) (right). Green arrows indicate a 1.5‐cm lesion between the head and uncinate process consistent with a neuroendocrine tumor.

**Figure 5:**
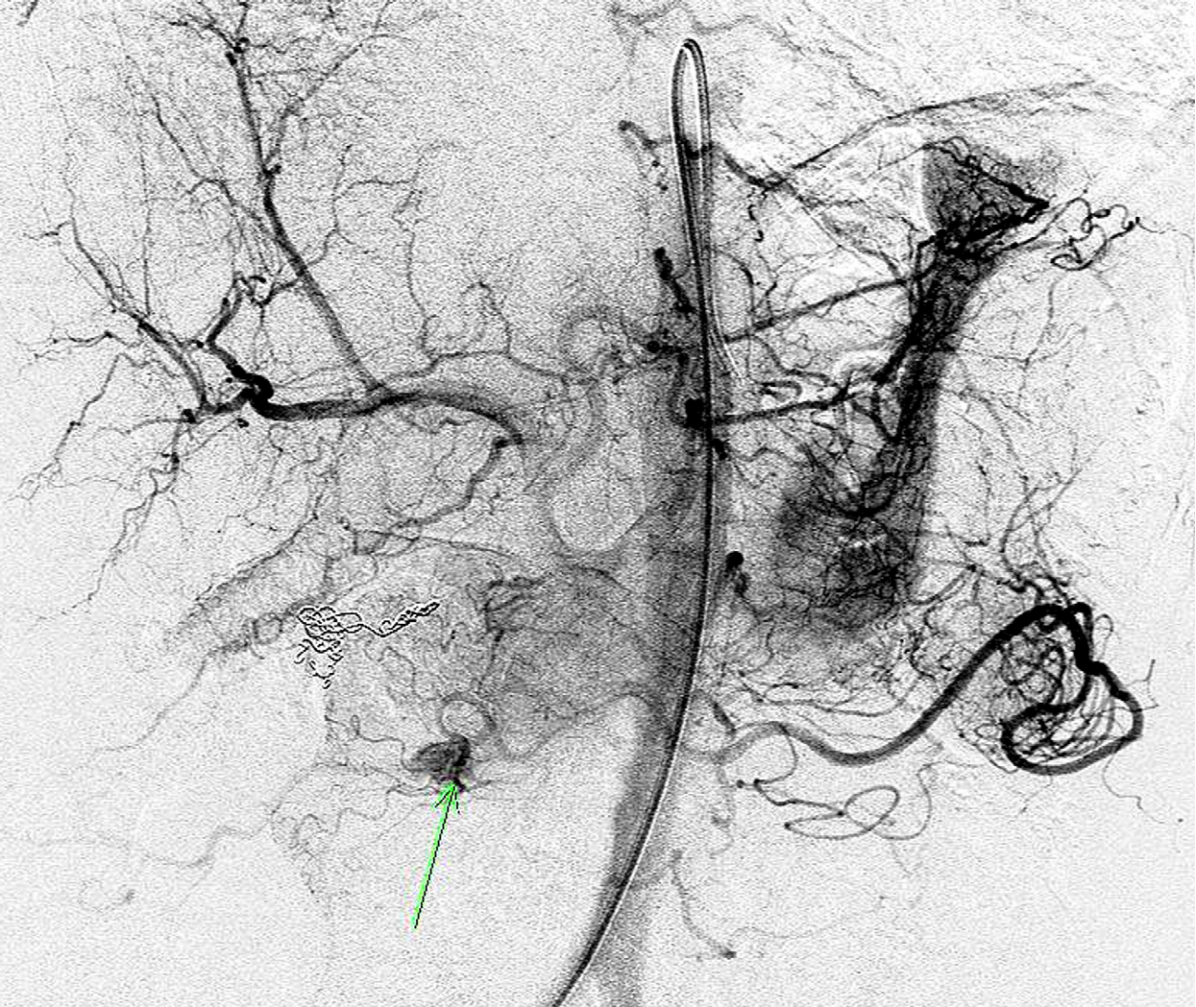
Pancreatic angiogram revealing an oval 8 mm diameter vascular blush, indicated by a green arrow, within the inferior aspect of the pancreatic head adjacent to the junction of D2/D3, therefore correlating with cross‐sectional imaging.

Currently no single imaging modality has sufficient sensitivity and specificity to unequivocally identify insulinomas 8. Therefore, several imaging modalities were combined in our patient to confirm the lesions’ location. Pancreatic CT demonstrated a 15‐mm enhancing lesion between the pancreatic head and the uncinate process (Fig. 4). Somatostatin receptor expression (typical in neuroendocrine tumors) was confirmed by Gallium‐68 DOTOTATE positron emission tomography/computed tomography (PET/CT) scan (Fig. 4). Pancreatic angiography revealed an anatomically concordant vascular blush, typical of a neuroendocrine tumor (Fig. 5). Venous sampling during angiography (ASVS) demonstrated a >2‐fold increase in hepatic vein insulin concentration following calcium stimulation, which confirmed the functional presence of an insulinoma in the uncinate process supplied by the superior mesenteric artery.

**Figure 6:**
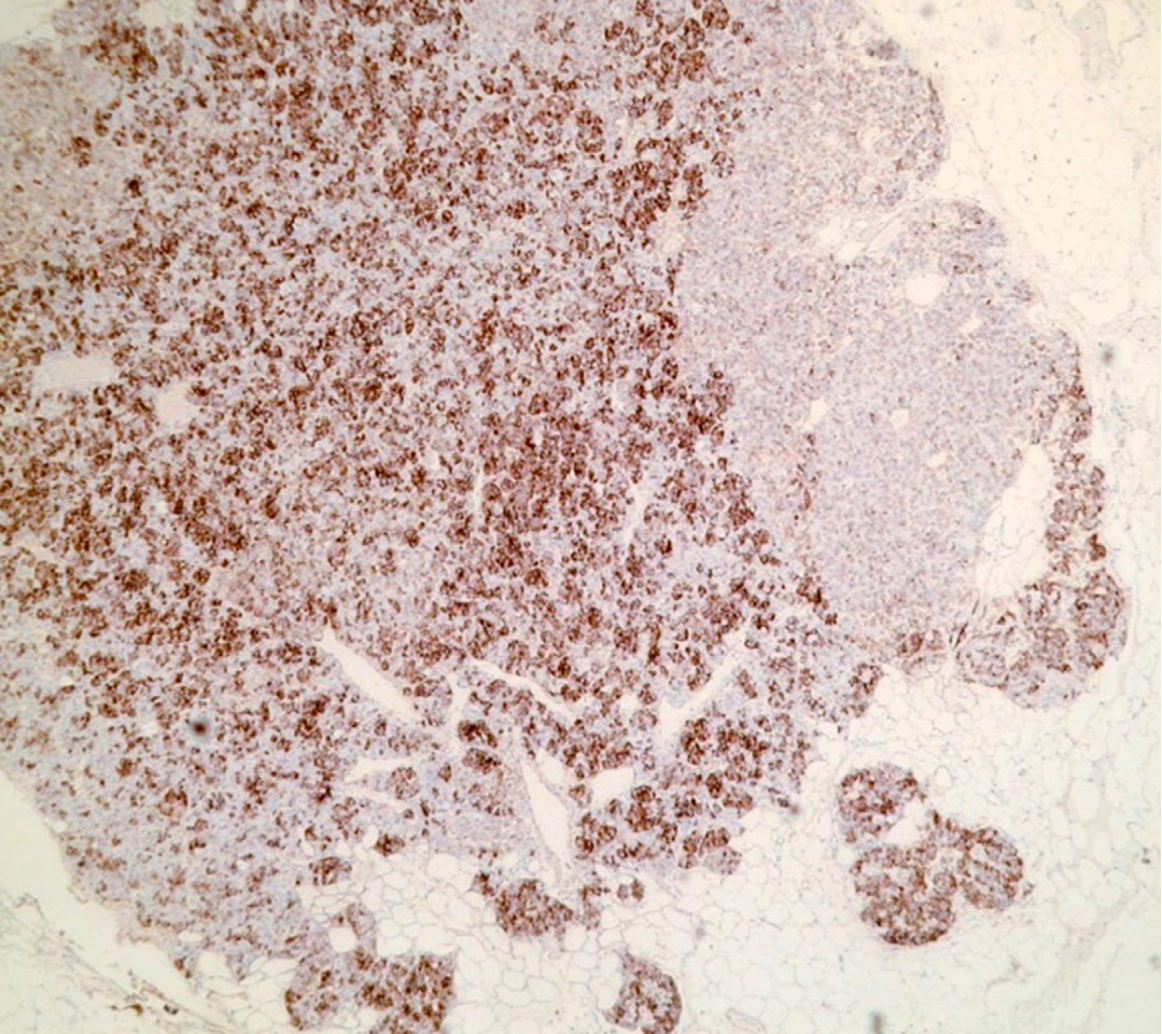
Light microscopy image of a tumor section demonstrating positive immunohistochemical staining for insulin (dark brown).

Medical management involved subcutaneous octreotide (a somatostatin analogue), and the patient commenced a diet consisting of small regular meals rich in complex carbohydrates to provide a sustained glucose source. Diazoxide is an effective inhibitor of pancreatic insulin secretion; however, it is contraindicated in renal failure so could not be used in this case. The gold standard management of an insulinoma is surgical enucleation 8, which was subsequently performed during an open laparotomy. Immunohistochemical analysis confirmed a well‐differentiated neuroendocrine tumor with histological features consistent with an insulinoma (Fig. 6). There was no vascular or perineural invasion, and Ki67 proliferation index was 1% indicating a low‐grade tumor.

The procedure was uncomplicated, and no further episodes of hypoglycemia occurred postoperatively. Given that she may have had a predisposition to developing insulinomas as a result of her tuberous sclerosis, she was followed up 3 months postsurgery and then annually as per guidelines 9.

## Discussion

Tuberous sclerosis complex is an autosomal dominant disorder with a prevalence of 1 in 10,000, that is characterized by the presence of hamartomas in multiple organs 2. TSC is caused by a loss‐of‐function mutation in one of two tumor suppressor genes. *TSC1* encodes hamartin, and *TSC2* encodes tuberin, which together form a complex that inhibits mTOR signaling (a crucial growth and proliferation regulatory pathway). Insulinomas are rare tumors with an annual incidence of 1 in 1,000,000, but nevertheless represent the commonest neuroendocrine tumor of the pancreas 8. The association of insulinoma and TSC was first described in 1959 and has been reported in a few subsequent cases (Table 1) 3, 4, 5, 6, 7. TSC has also been associated with other endocrine tumors including carcinoids, pheochromocytomas, and pituitary adenomas, but it remains unclear if insulinomas or other neuroendocrine tumors should be considered a feature of TSC, and current guidelines do not recommend routinely screening for neuroendocrine tumors in TSC patients 2. Thus, there is a need to share clinical experience through case reports and so potentially influence future guidelines. This case describes the oldest TSC patient with an insulinoma to date, and the coexistence of end‐stage renal failure represents an independent complicating factor in the diagnosis. In addition, we describe the investigation and management of endogenous insulin‐driven hypoglycemia according to best practice guidelines 1, 8, 9, 10, 11.

**Table 1:** Published cases of insulinomas diagnosed in the context of tuberous sclerosis complex

Publication	Demographics	Clinical features
Gutman & Leffkowitz 1959 3	24 year old female	Tonic–clonic seizures terminated by sugary drink. 3‐cm insulinoma excised from body of pancreas, leading to cessation of seizures.
Davoren & Epstein 1992 4	23 year old male	Tiredness and recurrent seizures. 3‐cm insulinoma excised from inferior portion of pancreatic head leading to cessation of seizures.
Kim et al. 1995 5	28 year old male	New behavioral changes with episodes of agitation and lethargy. 2‐cm insulinoma located in tail of pancreas. Partial pancreatectomy with resolution of symptoms.
Boubaddi et al. 1997 6	18 year old female	Symptomatic hypoglycemia secondary to insulinoma.
Eledrisi et al. 2002 7	43 year old male	Presented with confusion and slurred speech. Episodes of sweating and dizziness after prolonged fasting resolved with fruit juice. Large abdominal mass on palpation. 21‐cm insulinoma resected.

Tuberous sclerosis complex is associated with the formation of multiple benign tumors; thus, it is reasonable to hypothesize that there may also be a predisposition for developing insulinomas. In support of this hypothesis, molecular studies have shown that the TSC 1/2/mTOR signaling pathway can be aberrant in both sporadic TSCs (as the hamartin–tuberin complex is a critical negative regulator of mTOR) and neuroendocrine tumors (as central dysregulation of the mTOR pathway occurs in pancreatic neuroendocrine tumors 2). Thus, aberration in mTOR signaling is implicated in the molecular pathogenesis of both TSC‐related tumors and insulinomas, suggesting a common underlying molecular mechanism.

If the diagnosis of endogenous hyperinsulinism is established by laboratory investigations, localization studies to identify the causative lesion(s) should be undertaken. Our patient underwent a pancreatic CT and Gallium‐68 DOTATE PET/CT (Fig. 4), as well as a pancreatic angiogram with selective arterial calcium stimulation venous sampling (ASVS) (Fig. 5), with concordant findings. Gallium‐68 DOTOTATE PET/CT is a highly sensitive imaging modality for the majority of pancreatic neuroendocrine tumors; however, it is less effective in insulinomas being positive in less than one‐third of cases 8. Thus, the European Neuroendocrine Tumour Society (ENETS) Consensus guidelines recommend the use of multiple imaging modalities 8. MRI is more sensitive than CT in the diagnosis of insulinoma (74% vs. 65%) 10. Nevertheless, for preoperative surgical planning, CT is preferred to MRI as it is more effective at resolving important anatomical details such as vascular invasion and lymph node involvement 11. Other diagnostic modalities include endoscopic ultrasound (EUS) and functional localization by ASVS, which have reported sensitivities of up to 80.7% and 100%, respectively 8, 9. Insulinomas overexpress the glucagon‐like peptide 1 (GLP‐1) receptor, and GLP‐1 receptor analogue scintigraphy has recently proved a novel and sensitive imaging modality although currently not widely available 8.

Exogenous causes of hyperinsulinemia leading to hypoglycemia include insulin and sulphonylurea abuse. These were excluded by elevated C‐peptide levels and a normal sulphonylurea screen. Although an insulinoma is the most common cause of endogenous hyperinsulinemia, very rarely endogenous hyperinsulinemia can be caused by nesidioblastosis, a functional beta‐cell disorder, or insulin autoimmune syndrome whereby autoantibodies are directed against insulin or its receptor in patients naïve to exogenous insulin. Standard practice is to investigate these conditions if an insulinoma cannot be found 1.

Hypoglycemia is a common presentation in clinical practice; however, its subtle and varied symptoms can lead to delayed diagnosis. Presenting features of falls, confusion, and slurred speech are commonly encountered on the acute medical take as well as in a wide range of specialities involved in the long‐term management of patients with TSC. In this complex patient, there were multiple differentials for a presentation of slurred speech and confusion, which include factors related to TSC (e.g., cerebral hamartomas can lead to cognitive deterioration and seizures), end‐stage renal failure and dialysis (e.g., hemodynamic instability and sepsis). Interestingly, this patient developed hypoglycemia while on hemodialysis, despite the use of a glucose‐containing dialysate (1 g/L). Typically, glucose‐containing dialysates reduce the occurrence of hypoglycemia, although hypoglycemia can still occur especially in higher insulin states 12. This case emphasizes the need to consider and recognize hypoglycemia as an important differential diagnosis in such circumstances. It is particularly relevant in this case given the occurrence of recurrent symptoms without a clear explanation and the potential association of insulinomas with TSC.

This case highlights several important clinical points which are relevant to a wide range of specialities who frequently assess and manage patients with recurrent confusion and falls as well as patients with tuberous sclerosis. These include nephrologists, cardiologists, neurologists, as well as acute medicine, endocrine, respiratory, and elderly care physicians. Recurrent hypoglycemia should be considered in the differential diagnosis for all presentations of falls and confusion.

## Authorship

ANC, JHDB, JFT: conceived the study. ANC, AA, WSD, JDHB and JFT: investigated the patient and collected the data. ANC and LY: drafted the manuscript. ANC, LY, AA, WSD, JHDB, JFT: edited, reviewed, and approved the final version of the manuscript.

## Conflict of Interest

The authors declare that no conflict of interest exists.
